# Cortical tumor presenting with Parkinsonism

**Published:** 2015-10-07

**Authors:** Mi Song Choi, Bom Choi, Soo Jin Cho, Joo Yong Kim, Ki Han Kwon, Suk Yun Kang

**Affiliations:** ^1^ Department of Neurology, School of Medicine, Dongtan Sacred Heart Hospital, Hallym University, Hwaseong Si, Republic of Korea

**Keywords:** Parkinsonism, Brain Tumors, Magnetoencephalography, Motor Cortex, Primary Motor Area, Supplementary Motor Area

Parkinsonism is a syndrome with six major characteristics: Tremor at rest, rigidity, bradykinesia, loss of postural reflexes, flexed posture, and freezing.^1^ Parkinson’s disease (PD) is the most common form of Parkinsonism,^1^ but there are many other causes (i.e., drugs),^2^ clinician should be alert to alternative diagnoses, especially if patients with Parkinsonism have atypical findings for PD.

Parkinsonism due to brain tumor is very rare.^3^^,^^4^ Postulated mechanism was the compression of the basal ganglia.^3^ We would like to report an interesting case with intra-axial brain tumor presenting with Parkinsonism. In our case, the brain tumor did not compress the basal ganglia. We will discuss the possible mechanism.

A 55-year-old, right-handed man visited our hospital because he had a 1-month history of subjective motor weakness in the right extremities. He described that his handwriting became slow on writing long sentences and that he felt dragging of his right leg when walking for a long time. He said that he did not drag his foot at the beginning of the walk. He denied sudden onset and reported that the symptom had become worse. There were no vascular risk factors. There was no medication history. On detailed neurological examination, he was alert and fully oriented. There was no motor weakness. Mild sensory deficits for all modalities in the right extremities were seen. There was no cerebellar dysfunction. Deep tendon reflex was normal. Pathologic reflexes were not seen. He showed motor slowness in right finger tapping test and hand movements during the repetitive movements (bradykinesia). Rigidity was also observed on the right arm and leg. On his gait, there was no noticeable problem (i.e., hemiparetic gait, freezing, hesitation, etc.). There was no tremor. Brain magnetic resonance imaging (MRI) showed an enhancing mass lesion in the left paracentral area (Figure 1). We could not proceed further evaluation, because he wanted to go another tertiary hospital.

Our case illustrates two important clinical points. The first, the brain tumor in the paracentral area can cause Parkinsonism. The second, a high index of clinical suspicion is important for proper diagnosis and management, in particular, in the case that patients have unusual findings. In our case, unilateral sensory deficits and too short disease duration might raise suspicion of secondary Parkinsonism.

Parkinsonism caused by brain tumor is uncommon.^3^^-^^7^ Brain tumors showing Parkinsonism were various such as astrocytoma, meningiomas, craniopharyngiomas, and metastasis. They were usually supratentorial lesions involving the basal ganglia or the nigrostriatal tract, directly or indirectly.^3^^,^^6^ We think that the Parkinsonism of our patient may be associated with a brain tumor in the motor cortex. There is no direct involvement of the basal ganglia or no compression to the basal ganglia. Patients with Parkinsonism involving motor cortex were rarely reported, but the patients also had several unusual symptoms and signs, including headache, cognitive decline, motor weakness, and seizure.^3^^,^^6^ The tumors invaded other regions because the size was large or it was metastasis.^3^^,^^6^ Our patient was alert and did not complain any cognitive impairment. There was no definite motor weakness, normal tendon reflexes, and negative Babinski reflexes. The tumor was localized to the pericentral area.

**Figure 1:**
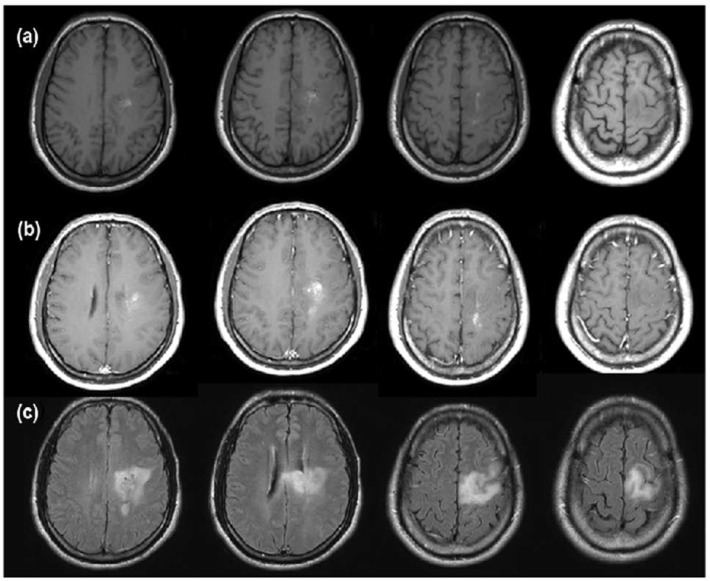
Brain magnetic resonance imaging (MRI) findings, (a) Axial T1-weighted; (b) axial, gadolinium-enhanced, T1-weighted; (c) axial fluid-attenuated inversion recovery. Brain MRI showed an enhancing infiltrating mass lesion in the left paracentral area (a, b), focal high signal is also noted on T1-weighted images, suggesting hemorrhage in the mass (a), MRI findings suggest malignant glial tumor such as malignant astrocytoma or malignant oligodendroglioma.

On initial neurological examination, it was hard to guess whether there was pyramidal tract involvement because there were normal tendon reflexes and negative Babinski reflexes.

The functional activity in the motor cortex is associated with bradykinesia and rigidity in PD.^8^^-^^10^ In generation of voluntary movements, the basal ganglia connection to motor cortical area is activated: The supplementary motor area (SMA) and the primary motor cortex (MI). The SMA is the main target of basal ganglia output and sends conspicuous projection to the MI. The role of SMA is known to prepare and execute the voluntary movement. The MI is associated with highly skilled movements.^8^ In 1-methyl-4-phenyl-1,2,3,6-tetrahydropyridine animal model, there was the disruptive neuronal activity of SMA and MI.^8^ It was reported that pyramidal tract involvement may contribute to bradykinesia. Pyramidal-tract type neurons showed abnormal firing rate in the parkinsonian monkey.^11^ The functional imaging studies showed altered activation of the motor cortex in PD.^9^^,^^10^ One study showed the hypoactivation of the motor cortex,^9^ and another, hyperactivation.^10^ These contradictory results may be due to different types of motor task.^9^^,^^10^ PD patients showed overall under activation of brain areas (including the motor cortex). The regional cerebral blood flow of these areas was positively correlated with a movement rate of the task.^9^ This hypoactivation may be explained by reduced thalamocortical output in PD.^12^ In the case of over-activation, it was explained with compensatory mechanism to decreased basal ganglia activity or reflection of rigidity, not bradykinesia.^10^

Our observations have some limitations. First of all, there is a lack of an appropriate follow-up. In fact, subacute onset or short duration of unilateral Parkinsonism is a strong red flag to the diagnosis of PD. We cannot exclude the possibility that my patient might be a patient with recent onset PD with an incidental brain mass on brain MRI, because we do not have any information about whether his Parkinsonism were improved after tumor treatment (i.e., tumor decompression), or after levodopa therapy. The improvement of his symptoms after levodopa treatment may suggest dopaminergic deficiency. Second, there might be argued that the description of parkinsonian features might not be enough, and we did not show his video clip, Unified PD Rating Scale, and dopamine transporter imaging, but unfortunately, these data are not available.

## Notes:

**How to cite this article: **Choi MS, Choi B, Cho SJ, Kim JY, Kwon KH, Kang SY. Cortical tumor presenting with Parkinsonism. Iran J Neurol 2015; 14(4): 219-21.
